# Navigating actions through the rodent parietal cortex

**DOI:** 10.3389/fnhum.2014.00293

**Published:** 2014-05-13

**Authors:** Jonathan R. Whitlock

**Affiliations:** Department of Neuroscience, Kavli Institute for Systems Neuroscience, Norwegian University of Science and TechnologyTrondheim, Norway

**Keywords:** parietal cortex, spatial navigation, cognitive motor function, rodent model, parieto-frontal network

## Abstract

The posterior parietal cortex (PPC) participates in a manifold of cognitive functions, including visual attention, working memory, spatial processing, and movement planning. Given the vast interconnectivity of PPC with sensory and motor areas, it is not surprising that neuronal recordings show that PPC often encodes mixtures of spatial information as well as the movements required to reach a goal. Recent work sought to discern the relative strength of spatial vs. motor signaling in PPC by recording single unit activity in PPC of freely behaving rats during selective changes in either the spatial layout of the local environment or in the pattern of locomotor behaviors executed during navigational tasks. The results revealed unequivocally a predominant sensitivity of PPC neurons to locomotor action structure, with subsets of cells even encoding upcoming movements more than 1 s in advance. In light of these and other recent findings in the field, I propose that one of the key contributions of PPC to navigation is the synthesis of goal-directed behavioral sequences, and that the rodent PPC may serve as an apt system to investigate cellular mechanisms for spatial motor planning as traditionally studied in humans and monkeys.

Direct neuronal recordings in PPC are conducted most often in either in awake, behaving monkeys or in rodents. Out of methodological necessity, primate subjects are typically head-restrained, completing tasks via precise movements of the hand, arm, or eyes. Rodent studies, on the other hand, often involve freely moving subjects solving navigational tasks. The dichotomous nature of behavioral tasks across species has produced largely divergent literatures, with monkey studies focusing on fine-grained analyses of cognitive functions such as decision making or movement planning, and rodent studies focusing largely on spatial navigation. While similarities in navigational mapping properties in PPC have indeed been demonstrated across species (e.g., Nitz, 2006; Sato et al., 2006), there is good hope for an even broader consilience of these oft-disparate literatures. Recent efforts have led to the development of tasks which isolate cognitive functions in rodents which hitherto have been studied more intensely in humans and monkeys, including visual attention (Broussard et al., 2006), movement planning (Erlich et al., 2011), working memory (Harvey et al., 2012), and decision making (Raposo et al., 2012a; Brunton et al., 2013). Such developments could lead to major advances in our understanding of the cellular underpinnings of cognitive abilities mediated by PPC, as the full arsenal of molecular and genetic tools applied in rodent models can be brought to bear on cognitive functions which for so long have been examined almost exclusively in higher primates.

In this Perspective, I highlight recent findings which further demonstrate similarities in the coding properties for planned motor behaviors in the rodent and primate PPC. Specifically, I propose that the representation of self-motion in PPC is consistent with the presence of action-goal modulation, a property described originally during complex reaching sequences in macaques, and that the rodent PPC computes navigational movement plans in a manner analogous to the prospective planning of spatially-targeted movements in monkeys. Though the focus here is on cognitive motor behaviors, PPC contributes spatial and abstract functions, discussed elsewhere, which are also likely instrumental to navigational behavior.

## A brief history of space in parietal cortex

The earliest evidence of spatial functions in parietal cortex came from clinical observations made by the Austro-Hungarian neurologist Balint (1909), where he observed that patients with bilateral damage to posterior parietal areas suffered visuospatial and visuomotor deficits, such as the inability to grasp an object held directly in front of the individual. The symptoms were neither strictly sensory nor motor, but appeared to result from a disconnection between the visual sensory system and the motor system for guiding eye and hand movements. Subsequent decades saw the identification of several additional functions in parietal cortex, including verbal memory, visual attention, and arithmetic ability in ventral regions, while dorsal PPC was thought to fulfill an all-purpose perceptual role in spatial processing (Critchley, 1953; Luria, 1966). The view of PPC as a sensory association area, which prevailed during much of the 20th century, was challenged fundamentally by the classic recording experiments of Mountcastle et al. in the 1975's, where they found that parietal neurons exhibited robust *motor* responses while monkeys performed visually-guided reaching tasks. The fact that a large proportion of the cells were selective for active, but not passive, movements suggested they were indeed bona fide elements of the motor command process (Mountcastle et al., 1975).

The next 20 years saw numerous recording studies in monkeys further revealing a role for parietal cortex in the early stages of spatially-guided motor planning (Andersen et al., 1997; Rizzolatti and Luppino, 2001). It was found that several sub-areas of PPC were robustly interconnected with frontal motor areas (Wise et al., 1997), and that such anatomically linked modules showed similar patterns of co-activation during the planning phases of instructed motor tasks (e.g., Johnson et al., 1996). Over the years it became clear that parietal cortex contained within it a patchwork of dedicated sub-areas which computed sensorimotor transformations specific to movements of the eye, hand or arm (Andersen and Buneo, 2002). The conceptual upshot of this collective body of work was the realization that spatial representation in many areas of parietal cortex served ultimately to enable targeted motor output, a view summarized effectively as “vision for action” (Goodale and Milner, 1992).

**Figure 1:**
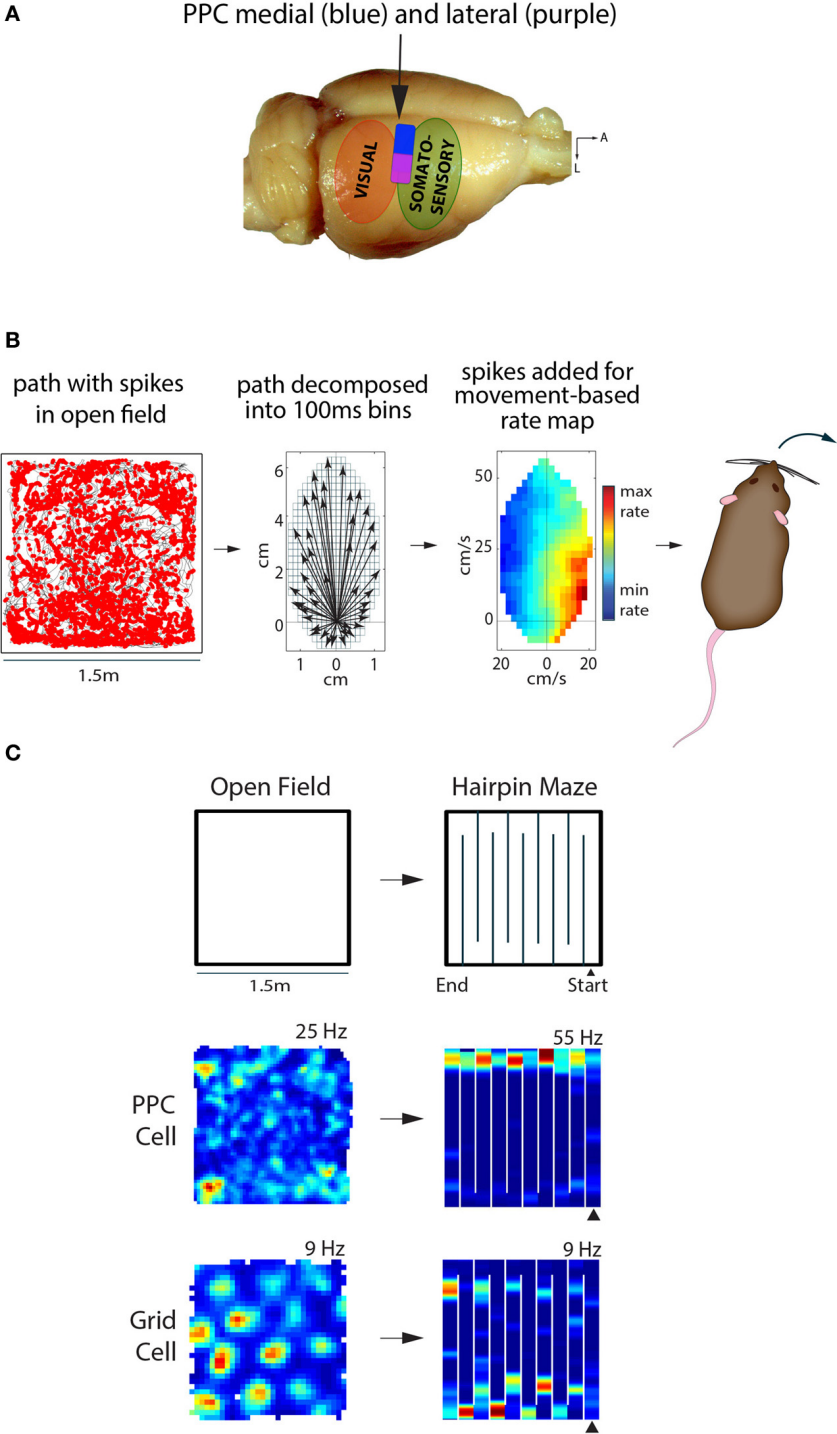
**Posterior parietal cortex (PPC) in the rat represents self-motion states, while entorhinal grid cells represent space. (A)** The rat PPC is located rostral to primary and secondary visual cortical areas, and caudal to somatosensory cortex (~3.5–5 mm posterior of Bregma). Medial PPC (~1.5–2.75 mm lateral of midline) is drawn in blue, while lateral PPC (~2.75–4.5 m lateral) is in purple; adapted from Paxinos & Watson “The Rat Brain,” 6th edition. **(B)** Left, the path of a rat foraging in an open arena is shown in black, with spikes from a PPC neuron overlaid as red dots. Middle, the animal's path is decomposed into movement vectors calculated in 100 ms time bins to resolve elementary linear and translational motion states during foraging; the schematic illustrates the population vector sum of all motion states during an open field recording session. Right, the firing rate of the PPC cell from the open field is visualized as a function of movement states, with hotter colors indicating higher firing rates and cooler colors indicating lower firing rates. This particular cell fired maximally when the animal made hard right turns. **(C)** Top, recordings sessions in the open field and hairpin maze were conducted in the same arena; walls were inserted into floor grooves under the open field to assemble the hairpin maze. Below, a representative example of a PPC cell showing no spatial tuning in the open field, but selective tuning to left turns in the hairpin maze. The MEC grid cell on the other hand showed clear, hexagonally-arranged firing fields in the open field; the hexagonal pattern broke down in the hairpin maze, but the cell still exhibited stable firing patters.

During this time, when so many laboratories were studying behavioral functions of the primate PPC, there was but one group recording from the homologous area in rats (McNaughton et al., 1989, 1994). The rodent PPC exhibits similar anatomical connections and cortical topology as seen in monkeys (Figure 1A; reviewed in Whitlock et al., 2008), though neural representations in the rodent PPC were characterized initially during navigational tasks not typically used with primates. This is because the first recordings in rodent PPC were conducted while experimenters were in the process of lowering microelectrodes aimed at the hippocampus to study place cells in an 8-arm radial maze (Chen and McNaughton, 1988). When trained animals ran in the maze it was found that 30–50% of PPC cells conjointly encoded certain movement types along with particular spatial trajectories, such that one cell fired during straight running toward the maze center, while another fired during clockwise turns at the outer ends of maze arms. Often times cells encoded two-part movement motifs such as straight running followed by left or right turns (McNaughton et al., 1994). More abstract spatial coding properties were later reported in PPC in a fascinating study showing the capacity of parietal neurons to track rats' progress along irregular, spatially-defined routes (Nitz, 2006). The main finding was that parietal neurons encoded route progress irrespective of spatial position or direction of motion, and the fact that they did so equally well in darkness or light implied a possible function in path integration. Based on the finding that PPC firing fields, unlike hippocampal place cells, scaled flexibly to match maze segments when they were lengthened or shortened, it was concluded that PPC cells were more tightly linked to the reference frame of the animals' route than a world-based spatial reference frame. A more recent study using *in vivo* calcium imaging and a virtual reality system also demonstrated the engagement of PPC cells during all phases of a virtual T-maze task, clearly suggesting a functional role for PPC not only in navigation, but in sensory processing and decision making (Harvey et al., 2012).

These studies and others, coupled with a large body of work demonstrating deficits in navigational tasks following lesions of PPC in rats, have been interpreted as evidence that PPC plays a role in spatial position coding based either on environmental landmarks or idiothetic (path integration-based) cues (Save and Poucet, 2009). In line with this view, it has also been shown that PPC contributes to the accurate updating of hippocampal place cell maps following local landmark displacement (Save et al., 2005). However, whether PPC and hippocampus interact primarily in the service of computing spatial maps remains unknown. While visual attention and effector position are almost invariably expressed in some type of spatial coordinate (Chafee and Crowe, 2013), it remains to be shown whether this manner of spatial representation plays any role in the very different process of allocentric spatial mapping. It has been shown, however, that PPC confers several functions likely play key roles in goal-directed navigation, including working memory (Harvey et al., 2012), visual and spatial attention (Broussard et al., 2006; Reep and Corwin, 2009), and the calibration of bodily movements against visual landmarks (McNaughton et al., 1994).

Until recently, the question of whether PPC and the hippocampal-entorhinal spatial system were coupled functionally had not been investigated, though evidence suggested that the two systems were capable of operating independently (e.g., Burke et al., 2005; Nitz, 2006, figure 3). Furthermore, the anatomical data showed that, while there are direct links between PPC and medial entorhinal cortex (MEC), the bulk of connectivity is indirect, possibly occurring via retrosplenial or presubicular cortices (Burwell and Amaral, 1998). Such issues motivated recent work to examine whether navigation-related representations in PPC were synchronized with spatial maps in the hippocampal-entorhinal circuit, using grid cell maps in MEC as a comparative benchmark.

## Parallel processing in parietal and entorhinal cortices

To determine whether representations in PPC and MEC were expressed synchronously or independently, it was necessary to record single units in tasks which elicited clear representations in both areas at the same time. We first tried open field recording experiments, but found that PPC cells lacked spatial selectivity (e.g., place fields). Instead, we found that firing rate maps computed in a reference frame based on self-generated motion states (Figure 1B, see also Chen et al., 1994) revealed robust tuning of PPC neurons to various forms of locomotion, such as turns to left or right at certain speeds. The tuning properties of PPC cells could be visualized in traditional space-based rate maps when animals ran in spatially structured tasks, such as a hairpin maze (Figure 1C). Critically, “remapping” experiments in which animals ran in similar hairpin mazes in different rooms showed that grid cells expressed totally different spatial maps across rooms, while PPC cells maintained the same tuning. This was the first direct demonstration that self-motion representation in PPC and spatial mapping in MEC occurred in parallel. Given that PPC neurons were insensitive to changes in spatial inputs *outside* the recording arena, we next tested whether they were sensitive to the structure of space *inside* the arena. We therefore compared the tuning properties of PPC in an actual hairpin maze and a “virtual” version of the task in which animals ran stereotypical north-south laps in an open arena. We found that PPC cells were tuned more strongly to the patterning of the animals' behavior than the spatial layout of the environment (Derdikman et al., 2009; Whitlock et al., 2012). The overall conclusion was that PPC cells coded self-motion states in a task-dependent manner, independently of spatial signaling in MEC.

## A case for cognitive motor functions in the rodent parieto-frontal pathway

**Figure 2:**
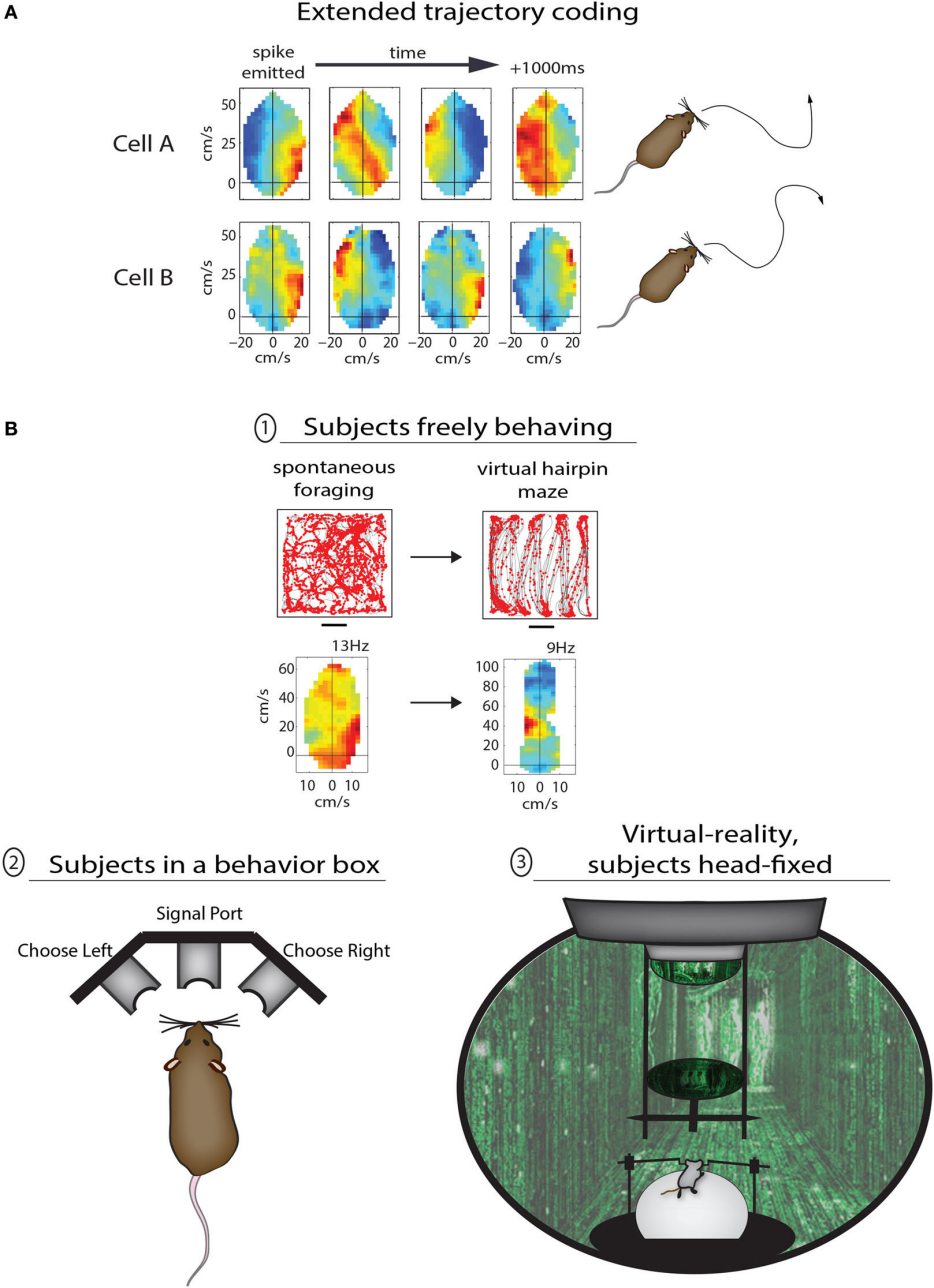
**Methods for studying the various functions of PPC in rodents. (A)** Two examples of PPC cells coding sequential trajectories during unrestrained foraging in the open field. *Cell A* fired when right turns were followed by sustained turning to the left; the motion sequence coded by the cell is illustrated to right (traced from open field data). *Cell B* fired bursts at the start of right-left-right-right movement sequences lasting more than 1 s. **(B)** Different levels of behavioral control for studying PPC in rodents. In the top example animals were freely foraging or performing stereotypical running sequences in the same open arena; the advantage to the lack of behavioral constraints here is the ability compare the coding properties of PPC neurons across very different modes of behavior. In example (2) the animal is confined to a behavior box with nose ports which afford the animal a means to trigger the delivery of stimuli and to provide a behavioral report. This type of apparatus is commonly used to study sensory decision making as well as motor planning, and offers the advantage of precise temporal control for cue presentation and behavioral assessment. The final example (3) shows a mouse in a virtual reality apparatus. This approach offers perhaps the highest degree of control by the experimenter, as the animals perform tasks while they are head-fixed and running on a track ball. One can therefore monitor precisely running speed and direction through any virtual environment.

The study above set out to characterize *spatial* mapping in PPC, but the results instead suggested a more primary sensitivity to the structure of *behavior*, and it is when one considers the representation of motor behaviors in PPC that additional conceptual links between the rodent and monkey come to light. One such example is the *prospective coding* of movements before they occur. Several fascinating lines of work in monkeys and humans have established that subsets of neurons in PPC as well as frontal motor areas become active prior to specific movements, regardless of whether the movement is eventually carried out or not (Wise et al., 1997; Andersen and Buneo, 2002; Desmurget and Sirigu, 2009). The pioneering experiments of Libet et al. (1982) demonstrated the existence of a “readiness potential” over the parietal lobe of human subjects more than 1 s before they moved their finger spontaneously to push a button, while recording studies in the primate PPC have demonstrated effector-specific preparatory signals prior to autonomously chosen eye or hand movements (Cui and Andersen, 2007). Several lines of recent research suggest that both parietal and premotor circuits in rodents are also engaged in prospective behavioral planning. In the case of Whitlock et al. (2012), PPC neurons coded not only ongoing movement states during spontaneous foraging, but on average showed tuning to movements 250 ms in advance, with subsets of cells coding multi-part trajectories more than 1 s ahead of time (Figure 2A). The virtual reality-based study by Harvey et al. (2012) showed that large-scale activity sequences played out in mouse PPC ensembles from the onset of left- and right-choice trials in the T-maze task (Figure 2B, bottom), demonstrating that the temporal structure of activity from one cell to the next could prove useful in predicting the navigational goal of the animal. Another study by Erlich et al. (2011) used extracellular recordings in the rodent premotor cortex during a memory-guided orienting task (Figure 2B, bottom) and found that more than 1/3 of neurons predicted the direction of head-orienting movements. A separate study by Raposo et al. (2012b) used a similar behavioral paradigm to study multi-sensory integration, and found that spiking activity in PPC was also predictive of the animals' behavioral report. In the coming years it will be of great interest to better understand how navigational and spatial movement plans are synthesized at the neuronal population level, and defining the causal function of upstream cortical inputs will likely be a major focus as well.

Another feature of motor representation in monkey parietal and premotor cortices is *action goal modulation*, in which actions are coded differently based on their final goal. This property was reported initially in parietal cortex (Fogassi et al., 2005) and later in premotor cortex (Bonini et al., 2010) of macaque monkeys performing 2-part grasping sequences with distinct goals—that is, they (i) grabbed a piece of food to either (ii) place it in a container or eat it. It was found that a narrow majority of neurons in both cortical areas showed different levels of activation during the initial grasping motion depending on the eventual goal of the trial, with some neurons coding “grasping to place” while other neurons coded “grasping to eat.” A conceptually similar phenomenon can be seen in Whitlock et al. (2012), in which PPC neurons showed completely different tuning depending on whether movement states occurred during random foraging or during goal-directed, structured sequences (Figure 2B, top). As opposed to providing a low-level readout of sensory or motor states, these data suggest that nested within movement representations are more abstract codes pertaining to task goal or working memory. Considering the prevalent representation of action goals in monkey premotor cortex, it is possible that task-specific coding of navigational behavior in PPC is driven by corollary discharges originating in frontal cortex (Taira et al., 1990).

Given that rodent experiments often involve freely-moving subjects, it raises the more general issue of whether rodent-based studies are controlled well enough to be comparable to primate studies. There are varying levels of control which can be applied during rodent behavioral experiments, each with advantages and disadvantages, ranging from free movement through open spaces, to operant conditioning in enclosed boxes, to head-fixation during virtual reality or other tasks (Figure 2B). The advantage of the first approach is the ability to compare neural coding properties of the same cells across very different modes of behavior, but such experiments are also the least controlled and are therefore perhaps better for studying more broadly congruent properties across species (as discussed above; Figure 2B, top). The introduction of a structured training apparatus, such as a T-maze or 3-port choice task (Figure 2B, bottom left) brings major advantages including precise timing and standardization of behavior. The latter type of task has been used recently by multiple groups to study motor planning (Erlich et al., 2011), decision making (Brunton et al., 2013), and multi-sensory integration (Raposo et al., 2012a) with a level of temporal precision more familiar to primate studies. Perhaps the most tightly-controlled behavioral paradigms in rodents utilize head-restraint, in which animals navigate in virtual environments or report sensory decisions using a track ball (Harvey et al., 2012; Sanders and Kepecs, 2012). Though this approach limits the types of behaviors which can be studied, they enable a rigorous level of control that would be necessary for detailed analyses of effector-specific movement planning (e.g., Cui and Andersen, 2007), or the representation of optic flow (e.g., Steinmetz et al., 1987). Very recent efforts have also succeeded in having rats voluntarily place their heads into kinematic mounts for 2-photon imaging of neural activity during the delivery of sensory stimuli or during decision making, and then removing their head between trials (Scott et al., 2013). Despite the recent advances, however, perfectly-matched experiments across monkeys and rodents will still be complicated by species-related differences in effector usage and ethology. Many of the basic questions relating to cognitive motor functions, though, are not necessarily specific to one species or another—such as the time course over which a movement plan evolves relative to action initiation, the cortical representation of movement goals, or the computational contribution of common anatomical pathways. While the monkey system presents phenomenal anatomical and functional segregation, the rodent preparation is favorable for applying opto- and pharmacogenetic methods which can be applied in a temporally precise manner to study the contribution of most any cortical sector or its anatomical afferents during a variety of cognitive behaviors (e.g., Sheppard et al., 2013).

## Conclusion

Many of the behaviors studied during neurophysiological experiments in the laboratory, such as reaching or grasping in the case of monkeys, can be thought of as solutions to problems which all species of animals must solve in order to survive. If a monkey is hungry, it must reach out to grab a piece of food; if a rat is hungry, it must run to a food source to eat—though the behaviors are not identical, comparable neural circuits must solve similar problems to achieve the common goal of eating. From an evolutionary perspective it can be argued that once a neural network solves a problem which leads to improved adaptive behavior, the structure of that network becomes perpetuated in the germ line. For example, place cells and grid cells were discovered first in rats but have since been described in homologous networks in every mammalian species tested, including humans (O'Keefe and Dostrovsky, 1971; Ekstrom et al., 2003; Hafting et al., 2005; Fyhn et al., 2008; Yartsev et al., 2011; Killian et al., 2012; Jacobs et al., 2013; Yartsev and Ulanovsky, 2013). This implies that place cells and grid cells date back at least 65–100 million years to the common ancestor of placental mammals (Meredith et al., 2011; O'Leary et al., 2013), and it is quite likely that such spatial circuitry arose long before in even simpler, more ancient organisms. The same principle applies to the parieto-frontal pathway, which is another common feature of all mammalian nervous systems. As opposed to generating spatial maps, it enables the synthesis of efficient movements and meaningful interactions with objects within those spatial maps, computing behavioral solutions to everyday problems which terrestrial vertebrates have encountered for countless millennia.

### Conflict of interest statement

The author declares that the research was conducted in the absence of any commercial or financial relationships that could be construed as a potential conflict of interest.
