# Corrigendum: Navigating actions through the rodent parietal cortex

**DOI:** 10.3389/fnhum.2017.00097

**Published:** 2017-02-28

**Authors:** Jonathan R. Whitlock

**Affiliations:** Department of Neuroscience, Kavli Institute for Systems Neuroscience, Norwegian University of Science and TechnologyTrondheim, Norway

**Keywords:** parietal cortex, spatial navigation, cognitive motor function, rodent model, parieto-frontal network

In the original article, we neglected to include the funder, the European Research Council, Starting Grant N° 335328 to JW, as well as funding from the Kavli Foundation, the Centre of Excellence scheme of the Research Council of Norway – Centre for Biology of Memory and Centre for Neural Computation, The Egil and Pauline Braathen and Fred Kavli Centre for Cortical Microcircuits and the National Infrastructure scheme of the Research Council of Norway – NORBRAIN. The author apologizes for this error and states that this does not change the scientific conclusions of the article in any way.

## Conflict of interest statement

The author declares that the research was conducted in the absence of any commercial or financial relationships that could be construed as a potential conflict of interest.

